# Mesoporous Transition Metal Oxides for Supercapacitors

**DOI:** 10.3390/nano5041667

**Published:** 2015-10-14

**Authors:** Yan Wang, Jin Guo, Tingfeng Wang, Junfeng Shao, Dong Wang, Ying-Wei Yang

**Affiliations:** 1 College of Chemistry, International Joint Research Laboratory of Nano-Micro Architecture Chemistry (NMAC), State Key Laboratory of Inorganic Synthesis and Preparative Chemistry, Jilin University, 2699 Qianjin Street, Changchun 130012, China; E-Mail: wangy2011@jlu.edu.cn; 2 State Key Laboratory of Laser Interaction with Matter, Changchun Institute of Optics, Fine Mechanics and Physics, Chinese Academy of Science, Changchun 130033, China; E-Mails: guoj@ciomp.ac.cn (J.G.); wtfeng@sina.com (T.W.); shaojunfeng1984@163.com (J.S.); wangd@ciomp.com.cn (D.W.)

**Keywords:** supercapacitor, pseudo-capacitor, transition metal oxides, specific capacity, mesoporous materials

## Abstract

Recently, transition metal oxides, such as ruthenium oxide (RuO_2_), manganese dioxide (MnO_2_), nickel oxides (NiO) and cobalt oxide (Co_3_O_4_), have been widely investigated as electrode materials for pseudo-capacitors. In particular, these metal oxides with mesoporous structures have become very hot nanomaterials in the field of supercapacitors owing to their large specific surface areas and suitable pore size distributions. The high specific capacities of these mesoporous metal oxides are resulted from the effective contacts between electrode materials and electrolytes as well as fast transportation of ions and electrons in the bulk of electrode and at the interface of electrode and electrolyte. During the past decade, many achievements on mesoporous transition metal oxides have been made. In this mini-review, we select several typical nanomaterials, such as RuO_2_, MnO_2_, NiO, Co_3_O_4_ and nickel cobaltite (NiCo_2_O_4_), and briefly summarize the recent research progress of these mesoporous transition metal oxides-based electrodes in the field of supercapacitors.

## 1. Introduction

**Figure 1:**
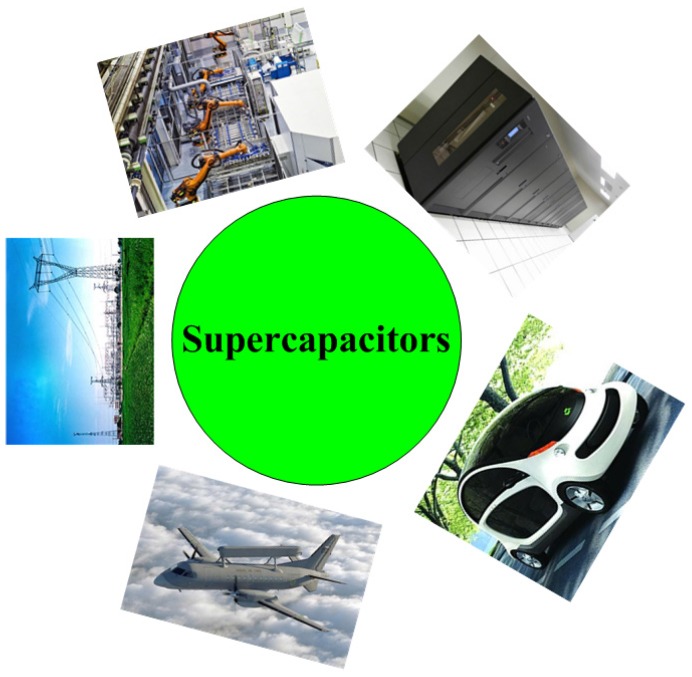
Some possible applications of supercapacitors in today’s society.

Energy crisis and environmental pollution have triggered the development of clean and renewable energy storage systems. Supercapacitors, also called electrochemical capacitors, are a novel type of charge energy storage devices in between traditional capacitors and batteries [1,2]. Compared with traditional capacitors, supercapacitors possess higher specific capacity and specific energy. Meanwhile, they exhibit higher specific power, shorter charging time, more efficient discharging than batteries, and they cause no pollution in the environment [3]. Although their energy density is 10 to 50 times lower than lithium ion batteries now, supercapacitors with a near unlimited cycle life are useful in the fields of power system, memory storage, and vehicle assistant equipment (Figure 1). For example, combining a supercapacitor and a battery in a single unit can create an electric vehicle with longer life, lower costs and more power. Based on the principle of energy storage, supercapacitors can be classified into electric double layer capacitors (EDLCs) and pseudo-capacitors [4]. EDLCs store charges using the very thin double layer structure formed at the interface between electrode and electrolyte, while pseudo-capacitors use fast and reversible redox reactions on the surface and bulk near the surface of electrodes for energy storage. Compared with EDLCs, pseudo-capacitors exhibit higher capacitance and higher energy density [4,5,6].

Nanoporous carbons are considered as one kind of ideal electrode materials for the EDLCs due to their large surface area, controlled pore structure, and high conductivity [7,8,9]. While for pseudo-capacitors, transition metal oxides as well as conducting polymers are common electrode materials [3]. Among them, transition metal oxides, such as ruthenium oxide (RuO_2_), manganese dioxide (MnO_2_), nickel oxides (NiO) and cobalt oxide (Co_3_O_4_), are investigated widely as electrodes materials [10,11,12]; both faradaic and non-faradaic mechanisms are involved in charge storage [13,14,15,16]. RuO_2_ involves faradaic charge-transfer reactions, and its cyclic voltammogram (CV) shape is quite broad, exhibiting a quasi-rectangular shape [17]. The good conductivity, rapid proton transport and larger surface area all contribute to rapid and reversible faradaic reactions with high capacitance [18]. Unfortunately, the high cost of RuO_2_ limits its large-scale applications. Therefore, significant efforts are being focused on finding a promising alternative to RuO_2_. MnO_2_, NiO, and Co_3_O_4_ are investigated as electrode materials to replace RuO_2_. MnO_2_ possesses low cost and rectangular voltammogram due to fast and reversible faradaic reactions [19,20], which is taken as one of the promising alternatives to RuO_2_. NiO, and Co_3_O_4_ are battery materials in the bulk state. When storage sites are limited on the surface of materials through nanostructure, significant pseudo-capacitance emerges [17]. Moreover, nickel cobaltite (NiCo_2_O_4_) also exhibits high capacitance values at short charge-discharge times recently, and redox behaviors of both nickel and cobalt are involved in energy storage [21,22]. Herein, RuO_2_, MnO_2_, NiO, Co_3_O_4_ and NiCo_2_O_4_ are specially selected, and their roles in supercapacitors will be reviewed.

A supercapacitor consists of electrode, electrolyte and separator, where electrode plays a key role for the performance of supercapacitor. It is a crucial task to explore electrode materials with excellent performance. According to the work principle of pseudo-capacitors, electrode materials and electrolyte should contact effectively, meanwhile, ions/charges should transport fast in the bulk of electrode and at the interface between electrode and electrolyte in order to achieve excellent properties. Therefore, transition metal oxides with mesopores (between 2 and 50 nm) are favorable for building supercapacitors with high performance due to their large specific surface area and suitable pore size distribution [23]. During the past decade, mesoporous RuO_2_, MnO_2_, NiO, Co_3_O_4_, and NiCo_2_O_4_ have been widely studied for supercapacitors. The simple and effective synthetic routes were developed, the nanomaterials with different morphologies were prepared, and the specific capacity and stability of devices were significantly improved. In this review, we briefly summarize the recent progress on these mesoporous transition metal oxide-based electrodes in the field of supercapacitors.

## 2. Transition Metal Oxides for Pseudo-Capacitors

### 2.1. Ruthenium Oxide (RuO_2_) for Pseudo-Capacitors

**Figure 2:**
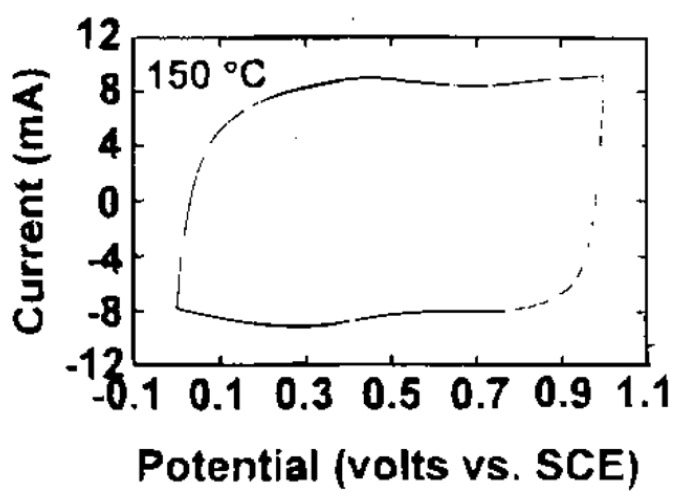
Cyclic voltammogram (CV) of RuO_2_·*x*H_2_O electrodes annealed at 150 °C. The voltage scan rate was 2 mV·s^−1^, and the electrolyte was 0.5 M of H_2_SO_4_ [18].

RuO_2_ is known as the best electrode material due to its large specific capacitance (700 F·g^−1^), low resistivity, high chemical and thermal stability [18,24,25]. When RuO_2_ is used as an electrode material, a series of redox processes occur, resulting in the variation of oxidation state among Ru^4+^, Ru^3+^ and Ru^2+^, where pseudo-capacitance mainly contributes to capacitance. Another feature is that these redox processes of RuO_2_ are reversible. These unique electrochemical features result in quasi-rectangular shape CV curve (Figure 2). Due to these remarkable advantages, RuO_2_, especially mesoporous RuO_2_, has attracted much attention from scientific community and industry. Until now, several different routes have been developed for the synthesis of mesoporous RuO_2_. For example, Galizzioli and Rochefort synthesized RuO_2_ by thermal decomposition of RuCl_3_ on metallic supports in solutions of electrolytes [26,27]. Zheng *et al.* prepared hydrous RuO_2_ by a sol-gel process and obtained specific capacitance as high as 720 F·g^−1^ for a powder formed at 150 °C [18]. This is the highest specific capacitance for RuO_2_. In addition, Liu *et al.* prepared RuO_2_ films by thermal and electrochemical methods [28]. Dubala and coworkers reported a surfactant-less and binder-free chemical bath deposition method to synthesize RuO_2_ thin films [29]. Although RuO_2_ has large specific capacitance, it is difficult to use RuO_2_ in real application, considering its very high cost and environmental toxicity. These limitations can be partly overcome by hybridization of RuO_2_ with other common conductive materials. For instance, NiO/RuO_2_ composite materials were prepared and a maximum specific capacitance of 210 F·g^−1^ was obtained for composite electrode with 10 wt % RuO_2_ in the voltage range of −0.4 to 0.5 V in 1 M KOH solution [30]. SnO_2_/RuO_2_ composite films were prepared by chemical bath deposition method. The specific capacitance of 150 F·g^−1^ was obtained by optimizing synthetic conditions [31]. RuO_2_/polyaniline electrodes were prepared by electrodeposition, revealing a specific capacitance of 474 F·g^−1^ and a small charge transfer resistance of 2.24 Ω [32]. RuO_2_/TiO_2_ nanotubes composites were synthesized by loading various amounts of RuO_2_ on TiO_2_ nanotubes and a maximum specific capacitance of 1263 F·g^−1^ was obtained [33]. By using carbon fibre paper as a support for RuO_2_, a specific capacitance of 977 F·g^−1^ was also obtained in a supercapacitor with 1M H_2_SO_4_ as electrolyte [34]. The special capacitances of these hybrid materials are summarized in Table 1.

**Table 1:** The special capacitances of hybrid RuO_2_ materials.

Materials	Special Capacitance (F·g^−1^)
RuO_2_	720 [18]
NiO_2_/RuO_2_	210 [30]
SnO_2_/RuO_2_	150 [31]
RuO_2_/Polyaniline	474 [32]
RuO_2_/TiO_2_	1263 [33]
RuO_2_/Carbon fibre paper	977 [34]

### 2.2. Manganese Dioxide (MnO_2_) for Pseudo-Capacitors

**Figure 3:**
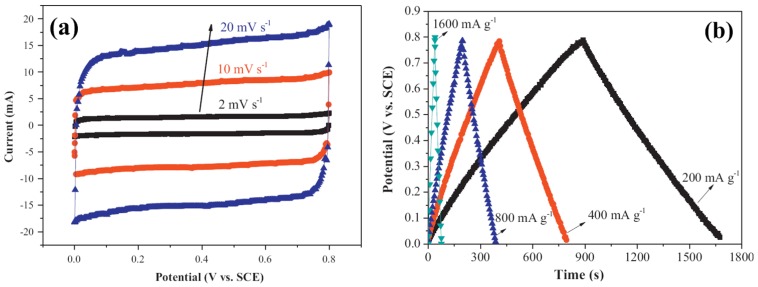
(**a**) CVs of MnO_2_ at scan rates of 2, 10, and 20 mV·s^−1^; and (**b**) Galvanostatic charge/discharges (GCDs) of MnO_2_ at current densities of 200, 400, 800, and 1600 mA·g^−1^ [41].

**Figure 4:**
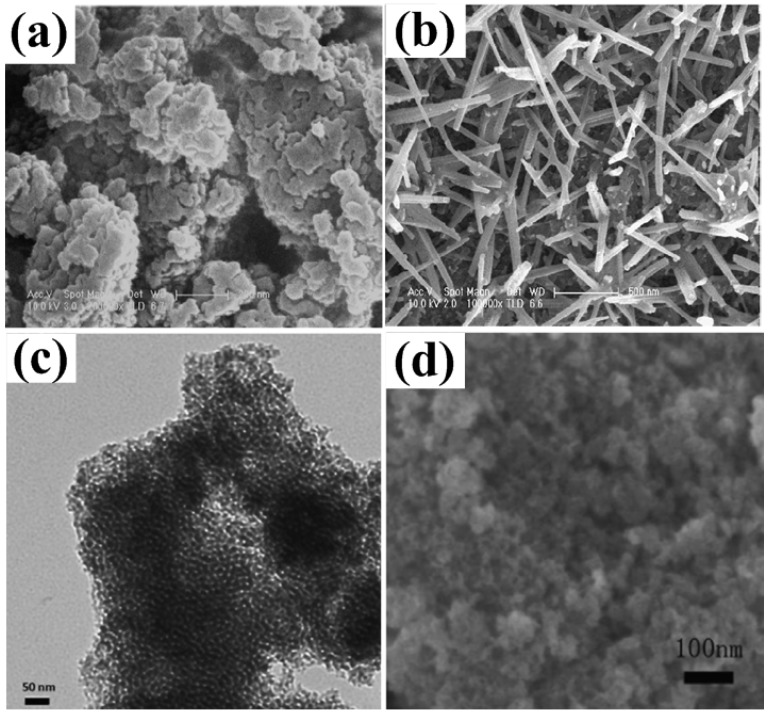
Scanning electron microscopy (SEM) images of MnO_2_ samples synthesized by sonochemical method with two different amplitudes: (**a**) 30 μm; (**b**) 60 μm [34]. Transmission electron microscope (TEM) (**c**) and SEM (**d**) images of mesoporous MnO_2_ synthesized through the reaction of potassium permanganate and ethanol under ultrasound irradiation [19].

MnO_2_ has been taken as a promising alternative to RuO_2_ because of its low cost and superior electrochemical performance [19,20,35,36]. Generally, capacitance properties of MnO_2_ are investigated in Na_2_SO_4_ electrolyte. In Na_2_SO_4_ electrolyte, reversible transitions of MnO_2_↔MnOONa occur, which is responsible for pseudo-capacitance properties [19,20]. The rectangular voltammogram implies fast and reversible capacitive behaviors, which, together with excellent cycle stability of MnO_2_, were demonstrated from the linearship of galvanostatic charge/discharge (GCD) profiles (Figure 3). According to theoretical calculation, specific capacitance of MnO_2_ is up to 1100 F·g^−1^ over a potential window of 1.0 V. However, the specific capacitances from experiments were five or ten times lower than theoretical values [37,38,39,40,41]. Both crystallographic forms (such as α, β, γ, δ, λ, *etc.*) and morphological nature are contributed to the low specific capacitance [37]. In order to obtain MnO_2_ with high specific capacitance, much effort has been devoted to the synthesis of mesoporous MnO_2_. Template method, microemulsion method, hydrothermal method, sonochemical method and ultrasound irradiation have been utilized to synthesize mesoporous MnO_2_ [38,39,40,41]. For example, semicrystalline gyroidal mesoporous MnO_2_ was prepared by using mesoporous silica KIT-6 as hard template, and stable reversible electrochemical behavior with capacitance of 220 F·g^−1^ in a potential range of −0.1–0.55 V was observed [38]. Mesoporous MnO_2_ nanoparticles with 4–5 nm of pore size were synthesized by a soft template method, and specific capacitance of 297 F·g^−1^ was obtained at a high loading level of 1.55 mg·cm^−2^ [39]. Mesoporous MnO_2_ with 2–20 nm of average pore sizes were obtained in sonochemical method from KMnO_4_ by using a tri-block copolymer (P123) as a soft template, and a maximum specific capacitance of 265 F·g^−1^ was obtained (Figure 4a,b) [40]. Mesoporous MnO_2_ with specific surface area of 192 m^2^·g^−1^ and 10 nm of pore distribution was synthesized through the reaction of potassium permanganate and ethanol under ultrasound irradiation (Figure 4c,d). The specific capacitance of 229 F·g^−1^ and the specific capacitance retention of 97.3% after 2000 cycles were obtained [19]. Birnessite-type mesoporous MnO_2_ nanospheres were synthesized by the microwave-hydrothermal method, and the electrochemical test showed that the specific capacitance was 210 F·g^−1^ at 200 mA·g^−1^ in 1.0 M Na_2_SO_4_ electrolyte with the specific capacitance retention and columbic efficiency of over 96% and 98%, respectively, after 300 cycles at 1.6 A·g^−1^ [41].

### 2.3. Nickel Oxide (NiO) for Pseudo-Capacitors

**Figure 5:**
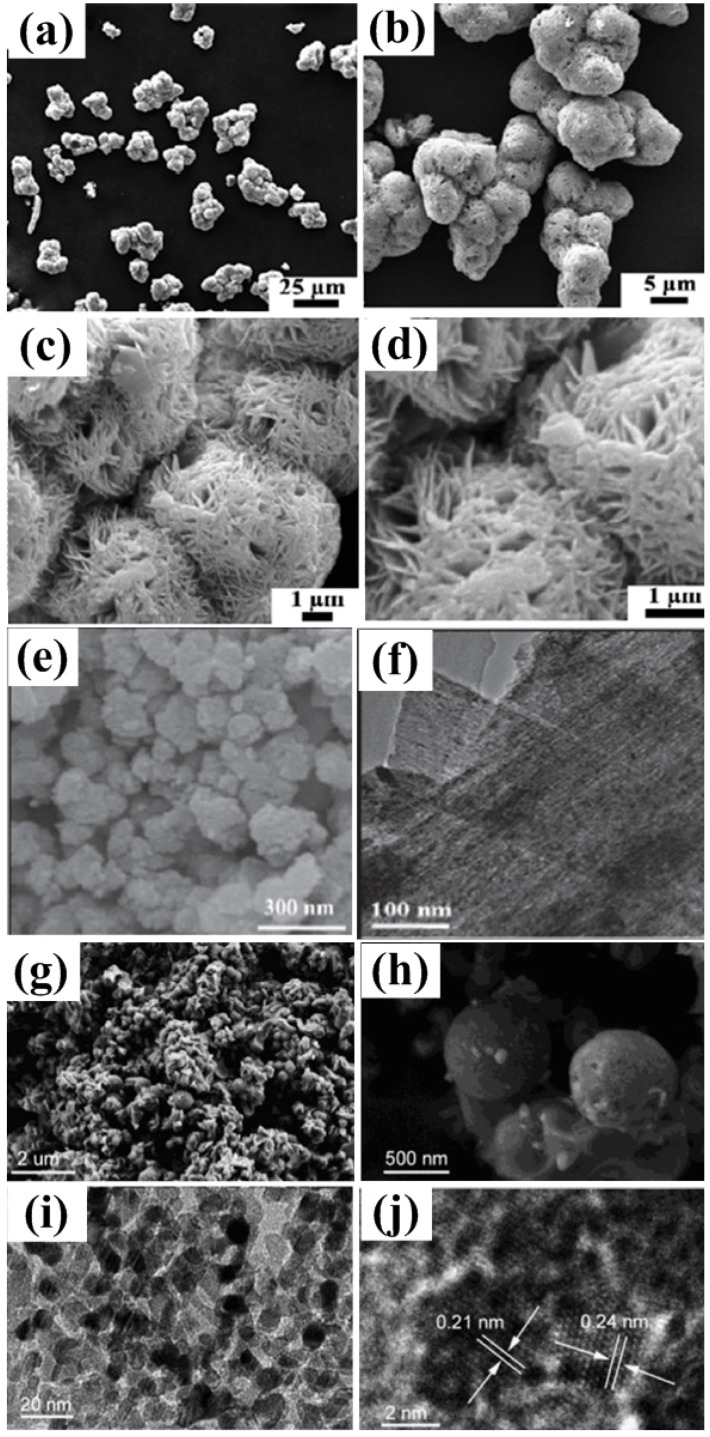
SEM images of the synthesized NiO microspheres (obtained by thermal decomposition of the Ni(OH)_2_ precursor after refluxing treatment for 60 min) at different magnifications (**a**–**d**) [43]. (**e**) SEM and (**f**) high resolution transmission electron microscopy (HRTEM) images of the NiO sample synthesized via a hydrothermal route with sodium dodecyl benzene sulfonate (SDBS) as an additive [46]. SEM images (**g**,**h**) at different magnifications, and TEM (**i**) and HRTEM (**j**) images of NiO sample prepared by using porous carbon nanospheres as a hard template [48].

NiO is regarded as a promising pseudo-capacitor material due to its high theoretical capacitance value of 2573 F·g^−1^, low cost and distinct redox [42]. For NiO, the variation of oxidation state of Ni is still not very clear. The reaction, NiO + OH ↔ NiOOH + e^−^, is usually believed to occur first. Then, the oxyhydroxide participates in the following reversible redox processes [17]. There are still some issues to be solved for the practical applications of NiO in supercapacitors. Among them, the significantly lower specific capacitance than theoretical value is one of the main issues. Due to the tight correlation between electrochemical performance of NiO and its porosity and surface area, most of the recent works are focused on the synthetic method and morphology study of materials. It has been known that mesoporous structured transition metal oxides are favorable for specific capacitance. On one hand, large specific surface is beneficial for electrolytes to access the electrochemically active sites. On the other hand, the mesoporous pore size is profitable for fast redox process. For the preparation of NiO with mesoporous structures, several methods have been employed. For example:

(1) Ni(OH)_2_ → NiO: Wu *et al.* prepared highly porous NiO via a combination of sol-gel process with supercritical drying method [5]. They prepared aerogel-like Ni(OH)_2_ samples and then heated them to achieve aerogel-like NiO. The as-synthesized NiO samples exhibited 80%–90% of porosity and 180.5–325.6 m^2^·g^−1^ of surface area. The average specific capacitance was observed to be *ca.* 75–125 F·g^−1^ between a potential window of 0–0.35 V *vs*. SCE [5]. Yuan and coworkers synthesized β-Ni(OH)_2_ microspheres by using coalescence and Ostwald-ripening mechanisms, and then these β-Ni(OH)_2_ microspheres were calcinated to produce hierarchical porous NiO microsphere (Figure 5a–d) [41]. Electrochemical data demonstrated that the hierarchical porous NiO nano/micro superstructures were capable of delivering a specific capacitance of 710 F·g^−1^ at 1 A·g^−1^ and offered a specific capacitance retention of *ca.* 98% after 2000 continuous charge-discharge cycles [43]. Lee *et al.* applied hexamethylenetetramine hydrolysis to synthesize Ni(OH)_2_ microstructures, followed by calcination to produce NiO microstructures at high temperature. The specific capacitance of fabricated NiO at 2, 5, 10, and 20 mV·s^−1^ scan rate was 718, 470, 420, and 403 F·g^−1^, respectively. The NiO microstructures had good retention for more than 1000 cycles in a cycling test [44].

(2) Hydrothermal Method: Li *et al.* used hydrothermal route to synthesize Ni(OH)_2_, and then prepared various mesoporous NiO hierarchical microspheres via thermal decomposition of Ni(OH)_2_ in air [45]. Electrochemical data demonstrated that the mesoporous NiO network-like hierarchical microspheres could deliver a specific capacitance of 555 F·g^−1^ at 2 A·g^−1^, and 390 F·g^−1^ even at a current density of 10 A·g^−1^ [45]. Yang *et al.* prepared mesoporous slit-structured NiO materials through a hydrothermal route with sodium dodecyl benzene sulfonate (SDBS) as an additive (Figure 5e–f). The as-prepared NiO samples presented specific capacitance of over 1700 F·g^−1^ in the potential range from 0.10 to 0.56 V at a constant current of 2 A·g^−1^, and capacitance retention of 90% after 1000 continuous charge-discharge cycles [46].

(3) Template Method: Mesoporous NiO was synthesized by a hydrothermal homogeneous precipitation method using mixed anionic/non-ionic surfactants as template. The electrochemical results showed that the as-prepared mesoporous NiO sample had a specific capacitance of 268 F·g^−1^ [47]. A nanospherical porous NiO electrode material was prepared by using porous carbon nanospheres as a hard template (Figure 5g–j). The GCD measurements demonstrated that the optimal electrode possessed a specific capacitance of 1201 F·g^−1^ at a discharge current density of 0.5 A·g^−1^ and cycling stability of 70% capacity retention after 500 continuous charge/discharge cycles [48].

(4) Microwave Assisted Heating Method: Meher *et al.* prepared porous NiO by microwave assisted heating method under homogeneous precipitation conditions [49]. Compared with that from the traditional reflux method, the sample prepared from microwave method showed higher rate specific capacitance (370 F·g^−1^) in the charge-discharge measurements made at a discharge current of 2 A·g^−1^ [49].

(5) Sol-Gel Method: NiO nanostructures with three distinct morphologies were fabricated by a sol-gel method, and nanoflower-shaped NiO with a distinctive three-dimensional (3D) network showed good supercapacitor properties [50].

**Figure 6:**
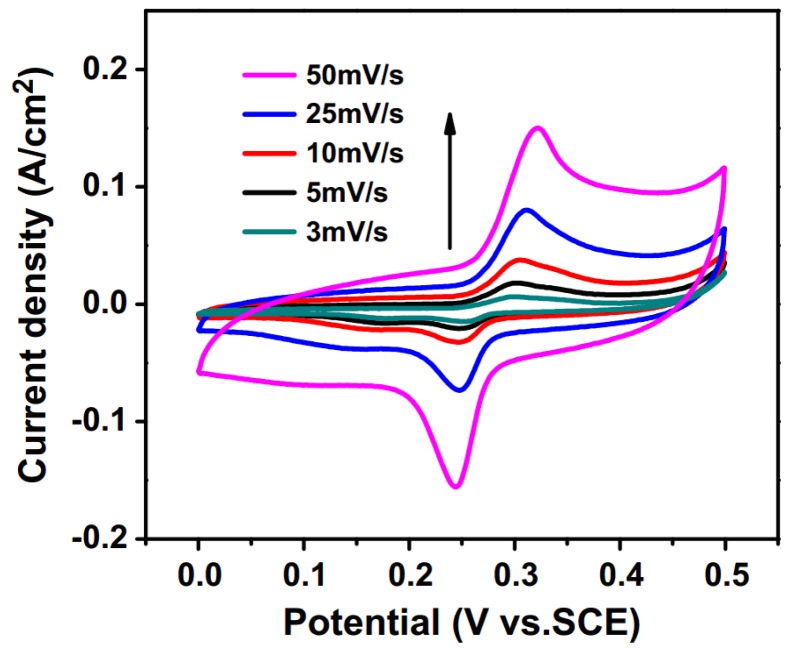
CVs of 1.50 mol% La^3+^-doped NiO electrode measured at scan rates from 3 to 50 mV·s^−1^ [51].

Another obstacle to specific capacitance of NiO is its poor conductivity, which results in low electron transport. To enhance electrochemical activity of the electrode, the dopants, such as transition and non-transition metal ions, have been introduced into the lattices of metal oxides. For instance, La^3+^ doped NiO microspheres with porous structures were fabricated using colloidal carbon spheres as hard template via a hydrothermal method followed by a calcination process. As a result, 1.5 mol% La^3+^ doped NiO showed a remarkable specific capacitance of 253 F·g^−1^ (two times higher than that of the pure NiO) and good cycling stability (34% capacity increase after 500 cycles) (Figure 6) [51].

There are also some reports on the synthesis of NiO/conductive materials. For example, monolithic NiO/Ni nanocomposite electrodes were fabricated recently, and the maximum capacitance was 910 F·g^−1^ due to the highly activated NiO surface layer and the conductive network of metal cores [52]. Flower-shaped NiO/α-Ni(OH)_2_ hybrid structures were synthesized by a solvothermal process. The hybrid composite demonstrates a solid capacitance value of 707 F·g^−1^ at 2 A·g^−1^ and 474 F·g^−1^ at a high discharge rate of 10 A·g^−1^. In order to further improve the performance and the capacitance retention, conductive carbon nanomaterials were used as supports for the NiO/α-Ni(OH)_2_ hybrid and it was found that single-walled carbon nanotubes significantly enhanced the performance of composite to 810 F·g^−1^ at a high current discharge of 10 A·g^−1^ [42].

### 2.4. Cobalt Oxide (Co_3_O_4_) for Pseudo-Capacitors

**Figure 7:**
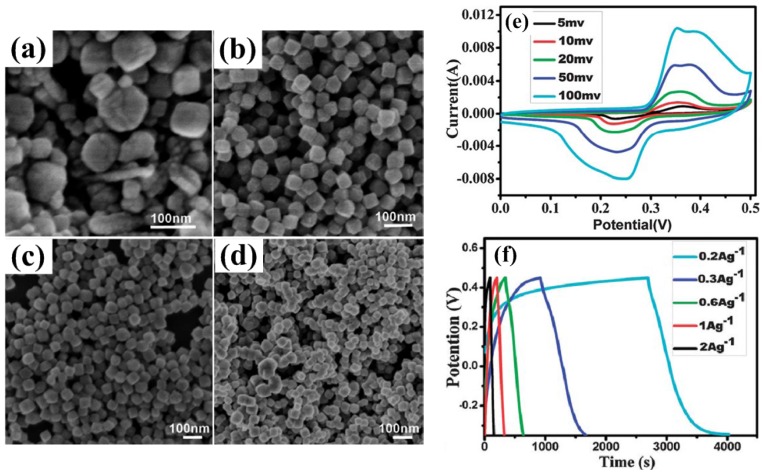
SEM images of Co_3_O_4_ samples obtained by adding different concentrations of mesoporous carbon nanorods: (**a**) 0 mg·mL^−1^; (**b**) 0.0175 mg·mL^−1^; (**c**) 0.025 mg·mL^−1^; (**d**) 0.05 mg·mL^−1^. (**e**) CV curves of the mesoporous Co_3_O_4_ nanocube electrode at scan rates of 5, 10, 20, 50 and 100 mV·s^−1^ and (**f**) galvanostatic charge/discharge (GCD) curves of the as-prepared electrode at different current densities [61].

Co_3_O_4_ has low environmental toxicity and high specific capacitance of 3560 F·g^−1^ in theory, and is of low cost [53,54]. During electrochemical processes, both pseudocapacitive behavior (CoOOH + OH^−^ ↔ CoO_2_ + H_2_O + e^−^) and battery-type behavior (Co_3_O_4_ + OH^−^ + H_2_O ↔ 3MOOH + e^−^) are contributed to capacitance, which results in high capacitance values (3560 F·g^−1^ in theory) [17,53,54]. However, the specific capacitances from real devices are much lower than that from theory. Electron effective transportation and ion fast diffusion would result in high performance of supercapacitors. Therefore, much effort has been devoted to synthesizing Co_3_O_4_ with appropriate nanostructures, particularly, Co_3_O_4_ nanomaterials with mesoporous structures, to improve the electron and ion transportation in electrodes and at the interface between electrode and electrolyte in supercapacitor devices [55,56]. For example, mesoporous nanocrystalline Co_3_O_4_ with a particle diameter of *ca.* 3 nm was synthesized based on strong chemical coordination interactions between Co^2+^ in solution and amino groups in the polyacrylamide template, and capacitance of 401 F·g^−1^ was obtained from these prerequisites [57]. Mesoporous Co_3_O_4_ microspheres with crater-like morphology were obtained by utilizing the mesoporous silica material named MCM-41 as a template. This material provided a specific capacitance of 102 F·g^−1^ and capacity retention of 74% in 500 continuous cycles test at a sweep rate of 3 mV·s^−1^ [58]. Hierarchically porous Co_3_O_4_ film was prepared by electrodeposition via liquid crystalline template, and specific capacitances of these Co_3_O_4_ films were 443 F·g^−1^ at 2 A·g^−1^ and 334 F·g^−1^ at 40 A·g^−1^, respectively [59]. Ultrafine nanosized Co_3_O_4_ materials with interconnected macroporous and mesoporous structure were synthesized through sol-gel method, followed by freeze-drying. The specific capacitance of the Co_3_O_4_ material was 742.3 F·g^−1^ at a scan rate of 5 mV·s^−1^ and the capacity retention was 86.2% after 2000 cycles [60]. With the assistance of mesoporous carbon nanorods, Co_3_O_4_ nanocubes with uniform diameter and high crystallinity were obtained. After calcination, mesoporous Co_3_O_4_ nanocubes were formed. Electrochemical tests revealed that the specific capacitance of Co_3_O_4_ nanocube electrode is *ca.* 350 F·g^−1^ at the current densities of 0.2 A·g^−1^ (Figure 7) [61]. In addition, meosoporous Co_3_O_4_ with other morphologies were also prepared. For example, tubular Co_3_O_4_ was fabricated by biomorphic synthesis route [62], nanosheets Co_3_O_4_ and microspheres Co_3_O_4_ were prepared by an ethanolamine-assisted solvothermal method and sequential thermal decomposition at mospheric pressure [63].

**Figure 8:**
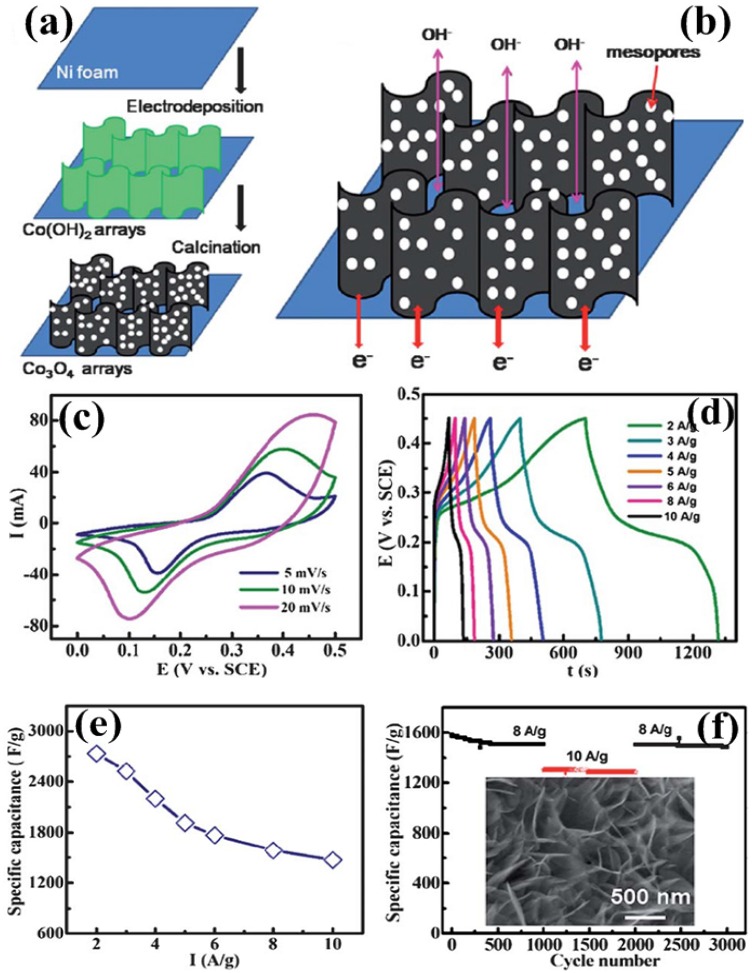
Schematic illustrations of (**a**) the general electrode design process; and (**b**) the application advantages of building 2D mesoporous Co_3_O_4_ ultrathin nanosheet arrays on Ni foam; (**c**) CV curves; (**d**) charge-discharge curves; (**e**) specific capacitance *versus* current densities; and (**f**) cycling performance of the Co_3_O_4_ nanosheet arrays/Ni foam electrode at varying current densities. The inset in (**f**) is field emission scanning electron microscope (FESEM) image of the Co_3_O_4_ nanosheet arrays/Ni foam electrode after cycling [64].

**Figure 9:**
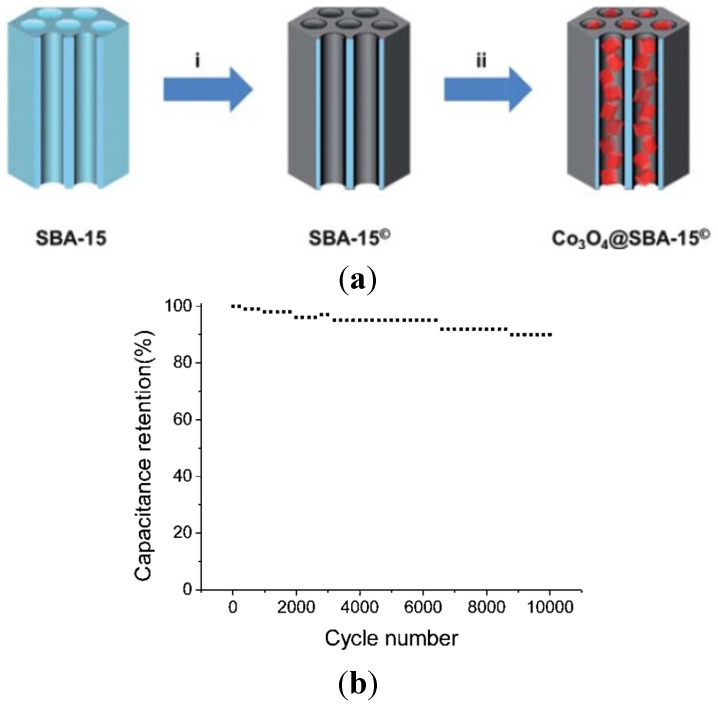
(**a**) Illustration of the preparation of Co_3_O_4_@SBA-15 supercapacitor electrode materials, showing (i) carbon nanomembrane (dark gray) formation on the walls (gray blue) of SBA-15 and (ii) incorporation of Co_3_O_4_ clusters into the cylinder type mesochannels and (**b**) cycle life of Co_3_O_4_(66%)@SBA-15 electrode material at a scan rate of 0.1 V·s^−1^ [65].

**Figure 10:**
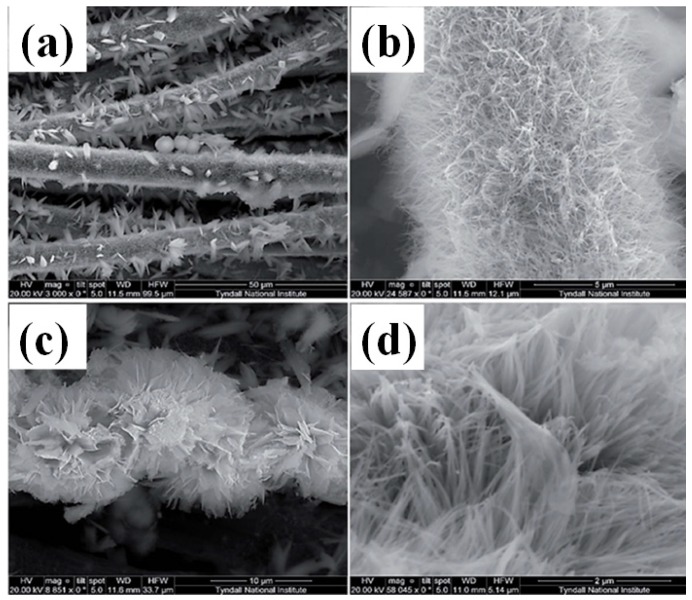
HRSEM images of Co_3_O_4_ nanowire/nanoflower on carbon fibre cloth at different magnifications [66]. (**a**) and (**b**) are the low magnification SEM images of bundles of carbon fibers; (**c**) and (**d**) are the high magnification SEM images.

In order to simplify the synthetic route and improve the electrochemical performance, mesoporous Co_3_O_4_ was grown on some support materials to form hybrid structures. For example, ultrathin mesoporous Co_3_O_4_ nanosheet arrays were grown on Ni foam with robust adhesion, which endows fast ion and electron transport, large electroactive surface area, and good structural stability. As a result, superior pseudo-capacitive performance was achieved with ultrahigh specific capacitance in the range of 2735–1471 F·g^−1^ and excellent cycling stability up to 3000 cycles (Figure 8) [64]. Co_3_O_4_ was embedded into SBA-15 nanoparticles to form composites by wetness impregnation method (Figure 9). The composite structure improves charged ion transmission inside the channels and the electrochemical utilization of Co_3_O_4_ during the charge/discharge processes. A supercapacitor electrode material based on Co_3_O_4_(66%)@SBA-15 exhibited a maximum specific capacitance of 1086 F·g^−1^ in 6 M KOH solution. After 10 000 cycles, retention of 90% of the initial capacitance was observed (Figure 9) [65]. Co_3_O_4_ nanowire/nanoflower hybrid structure on carbon fibre cloth was prepared via a hydrothermal approach followed by thermal treatment in air (Figure 10). A supercapacitor made from this hierarchical hybrid architecture showed a maximum specific capacitance of 4.8 mF·cm^−2^ at a constant density of 3 mA·cm^−2^ in organic electrolyte. In terms of energy and power, the supercapacitor conveyed an energy density of 4.2 mW·h·cm^−3^ with a power density of 1260·mW·cm^−3^ [66].

### 2.5. Nickel Cobaltite (NiCo_2_O_4_) for Pseudo-Capacitors

**Figure 11:**
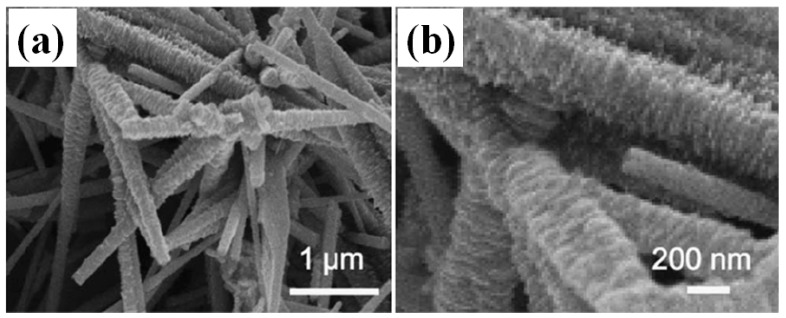
(**a**) and (**b**) FESEM images of the as-synthesized ultralayered mesoporous NiCo_2_O_4_ nanowires at different magnifications [70].

**Figure 12:**
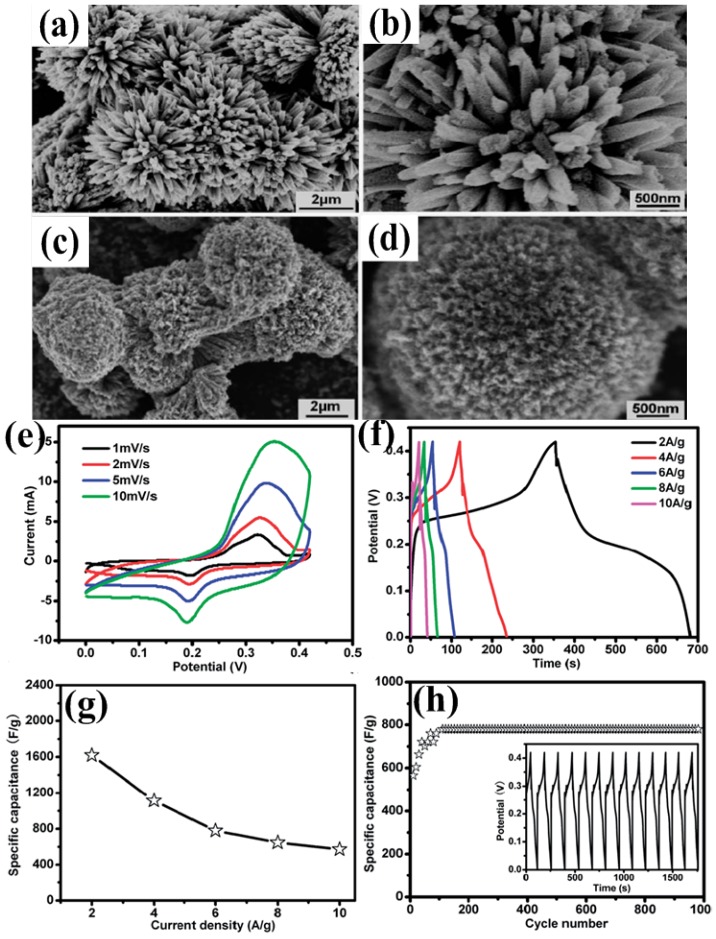
FESEM images of flower-like NiCo_2_O_4_ (**a** and **b**) and hierarchical mesoporous structured NiCo_2_O_4_ (**c** and **d**); electrochemical performance of hierarchical mesoporous NiCo_2_O_4_ electrodes. (**e**) CV curves at scan rates of 1.0, 2.0, 5.0 and 10.0 mV·s^−1^, respectively; (**f**) GCD curves at various current densities; (**g**) Effects of current density on its specific capacitance; (**h**) Cycling performance over 1000 cycles at a current density of 6.0 A·g^−1^. The inset shows the GCD curve at a current density of 6.0 A·g^−1^ [72].

From the above discussion, one can see that NiO and Co_3_O_4_ have attracted wide interest recently in supercapacitor applications due to their high theoretical capacity, abundant structure, good stability and low cost. However, in real supercapacitor devices, the observed specific capacitances are still far lower than theoretical values, especially at high rates [67]. A novel compound, NiCo_2_O_4_, attracts researchers’ attention. In 2010, Wei *et al.* obtained its specific capacitance as high as 1400 F·g^−1^ at a sweep rate of 25 mV·s^−1^ in a 1 M NaOH solution within a potential window of 0.04–0.52 V [68]. After that, NiCo_2_O_4_ has been extensively investigated for pseudo-capacitors. NiCo_2_O_4_ possesses good electrochemical activity and electrical conductivity at least two orders of magnitude higher than pure Co_3_O_4_ and NiO [69]. Both nickel and cobalt ions are involved in redox reactions, which is richer than pure nickel oxides and cobalt oxides. The energy storage occurs with reversible transformation of NiCo_2_O_4_ into nickel and cobalt oxyhydroxides as well as the reversible redox reaction of CoOOH + OH^−^ ↔ CoO_2_ + H_2_O + e^−^. The theoretical capacity for NiCo_2_O_4_, including the pseudocapacitive and the battery-like faradaic contributions, is 1203 C·g^−1^ or 2005 F·g^−1^ assuming at 0.6 V potential window in an aqueous alkaline electrolyte [22]. Up to now, NiCo_2_O_4_ with different structures and morphology, different dimensions have been reported and their electrochemical performances were improved greatly. For example, one-dimensional (1D) ultralayered mesoporous NiCo_2_O_4_ nanowires were synthesized by a template-free strategy (Figure 11). The ultralayered mesoporous nanowire electrode exhibited specific capacitance of 401 F·g^−1^ at 1 A·g^−1^ and excellent cycling stability (only *ca.* 10% loss after 5000 cycles) [70]. Mesoporous NiCo_2_O_4_ nanostructure was synthesized via a d-glucose-assisted solvothermal process. Electrochemical measurements showed that the spinel NiCo_2_O_4_ nanostructure heated at 300 °C exhibited maximum specific capacitances of 524 F·g^−1^ at 0.5 A·g^−1^ and 419 F·g^−1^ at 10 A·g^−1^ with good cycle stability and only ~9% of capacitance loss after 2500 cycles [71]. Hierarchical mesoporous spinel NiCo_2_O_4_ was synthesized by a hydrothermal method assisted by polyvinylpyrrolidone (PVP) and a post annealing treatment. Compared to conventional flower-like NiCo_2_O_4_, the hierarchical mesoporous structured NiCo_2_O_4_ exhibited better supercapacitance performance. The specific capacitance could reach 1619.1 F·g^−1^ at a current density of 2.0 A·g^−1^. When the current density was increased to 10.0 A·g^−1^, a specific capacitance of 571.4 F·g^−1^ could be obtained (Figure 12) [72]. Spinel NiCo_2_O_4_ was synthesized through a thermal decomposition method and specific capacitance was 764 F·g^−1^ at 2 mV·s^−1^ [73]. 3D network-like mesoporous NiCo_2_O_4_ nanostructures were fabricated through a solvothermal route coupled with a post annealing treatment. The as-obtained NiCo_2_O_4_ manifested specific capacitance of 931 F·g^−1^ at 3 A·g^−1^, capacity retention rate of 85.2 and 72.5% at 20 and 50 A·g^−1^, respectively [74].

In recent two years, the growth of NiCo_2_O_4_ on a conductive substrate attracts more and more attention. By this means, not only the conductivity of electrodes but also the specific surface areas is enhanced greatly. These unique composite electrodes may become ideal materials to improve the performance of pseudocapacitors. Several works have demonstrated the advantages of the composite electrodes. For example, mesoporous NiCo_2_O_4_ nanoneedles were grown on three-dimensional (3D) graphene-nickel foam and were then used to construct supercapacitor. The specific capacitance of NiCo_2_O_4_ nanoneedles was 1588 F·g^−1^ at 1 A·g^−1^ and the power density and energy density was 33.88 Wh·kg^−1^ at 5 kW·kg^−1^, respectively [75]. Hierarchical porous NiCo_2_O_4_ films composed of nanowalls on nickel foam were synthesized via a facile hydrothermal method. A capacity of 130 mA·h·g^−1^ was achieved at 2 A·g^−1^ with 97% capacity maintained after 2000 cycles [76]. NiCo_2_O_4_ multiple hierarchical structures composed of 1D nanowires and 2D nanosheets were grown on Ni foam. NiCo_2_O_4_ exhibited a specific capacitance of even up to 2623.3 F·g^−1^, scaled to the active mass of NiCo_2_O_4_ sample at a current density of 1 A·g^−1^. A nearly constant rate performance of 68% was achieved at a current density ranging from 1 to 40 A·g^−1^, and the sample retained approximately 94% of its maximum capacitance even after 3000 continuous charge-discharge cycles at a consistently high current density of 10 A·g^−1^ [77]. The ultrathin NiCo_2_O_4_ nanosheets supported on nickel foam were prepared via a two-step process, electrodeposition method followed with thermal treatment. The as-prepared samples were directly fabricated as electrodes for supercapacitors, and the outstanding electrochemical performance of 2517 F·g^−1^ was achieved at 1 A·g^−1^ and could still maintain 1200 F·g^−1^ at current density of 15 A·g^−1^. The cyclic stability was also tested under current density of 8 A·g^−1^, and 64% of initial capacitance was still maintained after 800 cycles [78].

**Figure 13:**
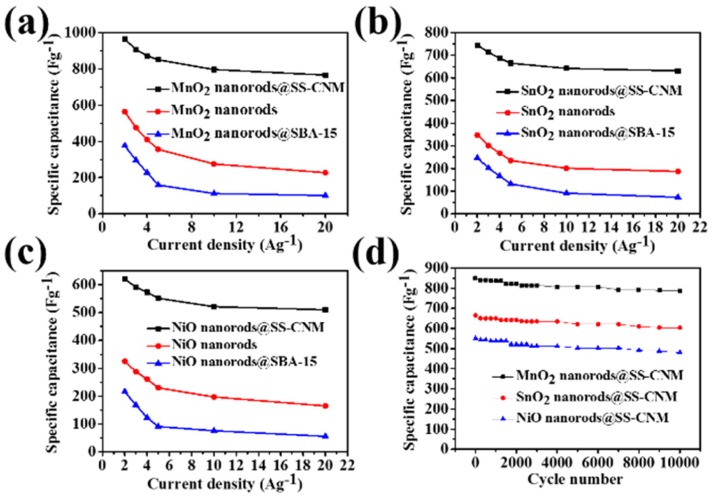
Curves of specific capacitance *versus* varied current density of MnO_2_ nanorods@SS-CNM (**a**); SnO_2_ nanorods@SS-CNM (**b**); and NiO nanorods@SS-CNM (**c**); (**d**) Cycle life of MnO_2_ nanorods@SS-CNM, SnO_2_ nanorods@SS-CNM, and NiO nanorods@SS-CNM electrode materials at a scan rate of 0.1 V·s^−1^ [79].

Although transition metal oxides, such as RuO_2_, MnO_2_, NiO and Co_3_O_4_, have been widely investigated for pseudo-capacitors, their practical applications are still limited due to their poor stability and low electrical conductivity [79]. While active carbon materials possess good conductivity and high stability. Thus, combining transition metal oxides with carbon materials may enhance the electric conductivity, and improve electrochemical performance of supercapacitors. Design and synthesis of transition metal oxides/active carbon materials were also reported [4,80,81,82,83,84,85]. For example, RuO_2_/ordered mesoporous carbon materials were prepared by impregnating an ordered mesoporous carbon CMK-3 with RuCl_3_·xH_2_O solution followed by annealing in nitrogen atmosphere. The annealing temperature and the RuO_2_ content had great influence on the specific capacitance of composites. The highest specific capacitance reached 633 F·g^−1^ by adjusting synthetic conditions [4]. Metal oxide nanorods (MnO_2_, SnO_2_, NiO) inside mesoporous silica supported carbon nanomembranes (denoted as SS-CNM) were applied for electrodes to fabricate symmetrical supercapacitors. Owing to the high electrical conductivity of SS-CNM and the intimate contact between the carbon membrane and well-ordered metal oxide nanorods, specific capacitances of MnO_2_ nanorods/SS-CNM, SnO_2_ nanorods/SS-CNM, and NiO nanorods/SS-CNM were achieved to be 964, 745, and 620 F·g^−1^, respectively. In addition, less than 10% of capacitance decays over 10 000 circles and energy density was 33.5, 25.7, and 21.6 Wh·kg^−1^ for MnO_2_ nanorods/SS-CNM, SnO_2_ nanorods/SS-CNM, and NiO nanorods/SS-CNM, respectively (Figure 13) [79]. Mesoporous NiO/reduced graphene oxide composites were synthesized by a hydrothermal route. Because the 3D graphene conductive network and the mesoporous structure were favorable for charge transportation and electrolyte diffusion, NiO/RGO composites exhibited high specific capacitance (1016.6 F·g^−1^) and good cycling stability (94.9% capacitance retention after 5000 cycles) [85].

## 3. Conclusions

Some recent achievements on mesoporous transition metal oxides (RuO_2_, MnO_2_, NiO, Co_3_O_4_ and NiCo_2_O_4_) for applications in supercapacitors were selected and reviewed. From this research, one can see that much progress has been made in the past decade. High specific capacity and excellent stability of nanomaterials have been observed from some mesoporous materials. In order to make the electrochemical performance of each material chosen in this mini-review more clear, their highest specific capacitance values and synthetic methods from literature mentioned in this paper are listed in Table 2. In the following research, simple synthetic methods that can produce metal oxide nanomaterials with appropriate morphology and are suitable for large-scale production should be a continuous concern for the purpose of real supercapacitor devices. In addition, attention should also focused on the design and synthesis of composite materials, such as transition metal oxides/metal, and transition metal oxides/active carbon, even on the combining of ionic liquids with mesoporous transition metal oxides. By doing so, the electrochemical performance of mesoporous transition metal oxide-based pseudo-capacitance could be envisioned to be improved substantially attributing to the synergistic effect of individual constituents. Especially, composite materials with 3D structures may provide more efficient and more rapid transportation for ions and electrons and thus result in higher electrochemical performance.

**Table 2:** The best special capacitances of each material.

Materials	Synthetic Method	Specific Capacitance (F·g^−1^)
RuO_2_	Sol-gel method	720 [18]
MnO_2_	Template method	297 [39]
NiO	Gydrothermal method	1700 [46]
Co_3_O_4_	Sol-gel method followed by freeze-drying	742.3 [60]
Co_3_O_4_/Ni	Robust adhesion	2735–1471 [64]
NiCo_2_O_4_	Sydrothermal method assisted by PVP and a post annealing treatment	1619.1 [72]
NiCo_2_O_4_/Ni	Hydrothermal method followed by annealing process	2623.3 [77]
RuO_2_/Order mesoporous carbon	Soak followed by annealing	633 [4]
MnO_2_/Mesopous silica supported carbon nanomembranes	*In situ* grown	964 [79]
NiO/Mesopous silica supported carbon nanomembranes	*In situ* grown	620 [79]
Co_3_O_4_/MWCNT	Chemical co-precipitation method	418 [80]
